# 3-Nitro-4-(propyl­amino)­benzonitrile

**DOI:** 10.1107/S1600536811045533

**Published:** 2011-11-12

**Authors:** Shang-Lian Liu, Hong-Sheng Jia

**Affiliations:** a Department of Biological and Chemical Engineering, Chien-shiung Institute of Technology, Taicang 215411, Suzhou, People’s Republic of China

## Abstract

In the title compound, C_10_H_11_N_3_O_2_, the nitro group is essentially coplanar with the aromatic ring [dihedral angle = 1.3 (3)°] and forms an intra­molecular amine–nitro N—H⋯O hydrogen bond. In the crystal, weak inter­molecular aromatic C—H⋯O_nitro_ hydrogen bonds link the mol­ecules. Weak aromatic ring π–π inter­actions [minimum ring centroid separation = 3.7744 (13) Å] are also present.

## Related literature

For the synthesis of the title compound, see: Ates-Alagoz & Buyukbingol (2001 ▶). For standard bond lengths, see: Allen *et al.* (1987 ▶).
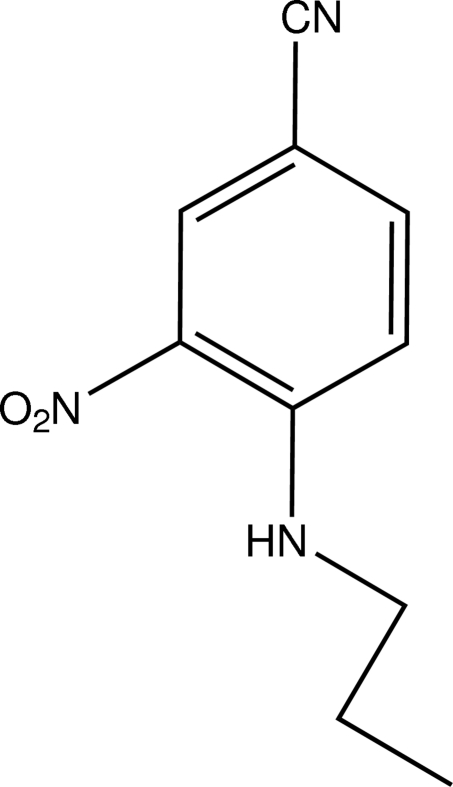


## Experimental

### 

#### Crystal data

C_10_H_11_N_3_O_2_
*M* *_r_* = 205.22
Triclinic, 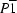

*a* = 7.6320 (15) Å
*b* = 7.9200 (16) Å
*c* = 9.2440 (18) Å
α = 109.30 (3)°
β = 91.28 (3)°
γ = 93.00 (3)°
*V* = 526.2 (2) Å^3^
*Z* = 2
Mo *K*α radiation
μ = 0.09 mm^−1^
*T* = 293 K
0.30 × 0.20 × 0.20 mm

#### Data collection

Enraf–Nonius CAD-4 four-circle diffractometer
Absorption correction: ψ scan (North *et al.*, 1968 ▶) *T*_min_ = 0.973, *T*_max_ = 0.982
2073 measured reflections
1918 independent reflections
1321 reflections with *I* > 2σ(*I*)
*R*_int_ = 0.017
3 standard reflections every 200 reflections  intensity decay: 1%

#### Refinement

*R*[*F* ^2^ > 2σ(*F* ^2^)] = 0.051
*wR*(*F* ^2^) = 0.165
*S* = 1.04
1918 reflections
137 parameters
H-atom parameters constrained
Δρ_max_ = 0.18 e Å^−3^
Δρ_min_ = −0.17 e Å^−3^

### 

Data collection: *CAD-4 Software* (Enraf–Nonius, 1994 ▶); cell refinement: *CAD-4 Software*; data reduction: *XCAD4* (Harms & Wocadlo, 1995 ▶); program(s) used to solve structure: *SHELXS97* (Sheldrick, 2008 ▶); program(s) used to refine structure: *SHELXL97* (Sheldrick, 2008 ▶); molecular graphics: *PLATON* (Spek, 2009 ▶); software used to prepare material for publication: *SHELXL97*.

## Supplementary Material

Crystal structure: contains datablock(s) global, I. DOI: 10.1107/S1600536811045533/zs2155sup1.cif

Structure factors: contains datablock(s) I. DOI: 10.1107/S1600536811045533/zs2155Isup2.hkl

Supplementary material file. DOI: 10.1107/S1600536811045533/zs2155Isup3.cml

Additional supplementary materials:  crystallographic information; 3D view; checkCIF report

## Figures and Tables

**Table 1:** Hydrogen-bond geometry (Å, °)

*D*—H⋯*A*	*D*—H	H⋯*A*	*D*⋯*A*	*D*—H⋯*A*
N1—H1*A*⋯O2	0.86	2.00	2.641 (2)	131
C9—H9*A*⋯O1^i^	0.93	2.42	3.331 (2)	165

Symmetry code: (i) 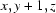.

**Fig. 1.**
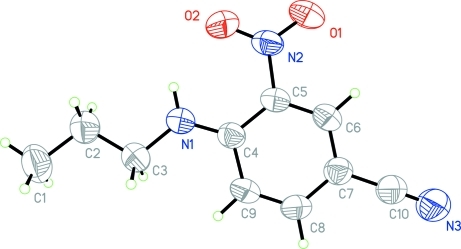
The molecular structure of the title compound showing the atom-numbering scheme, with displacement ellipsoids drawn at the 30% probability level.

**Fig. 2.**
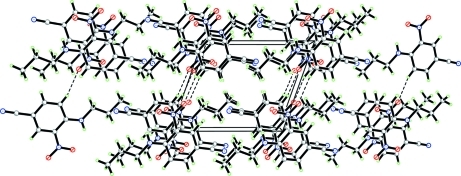
A packing diagram of the title compound, with intermolecular hydrogen bonds shown as dashed lines.

## supplementary crystallographic information

### Comment

We report herein the crystal structure of the title compound C_10_H_11_N_3_O_2_. In this molecule (Fig. 1), the bond lengths and angles (Allen *et al.*, 1987) are within normal ranges. The nitro group is essentially coplanar with the aromatic ring forming a dihedral angle of 1.3 (3)° with the ring. The amine H atom forms an intramolecular hydrogen bond with a nitro O-acceptor (O2) (Table 1). In the crystal structure, a weak intermolecular aromatic C—H···O_nitro_ hydrogen bond links the molecules (Fig. 2) while also present are weak aromatic ring π–π interactions [minimum ring centroid separation, 3.7744 (13) Å].

### Experimental

The title compound was synthesized using the procedure of (Ates-Alagoz & Buyukbingol, 2001). 4-Chloro-3-nitrobenzonitrile (4.2 g, 0.023 mol) was refluxed in 25 ml of *n*-propylamine and 50 ml of tetrahydrofuran for 4 h. Then solvents were evaporated, water was added to give a precipitate which was collected by filtration and washed with cold ethanol (2 x 15 ml) to afford the yellow solid (4.2 g, 89%). The pure title compound was obtained by recrystallizing from ethanol, with crystals suitable for X-ray diffraction obtained by slow room-temperature evaporation of an ethanol solution.

### Refinement

Hydrogen atoms were positioned geometrically, with C—H = 0.93 Å (aromatic), 0.97 Å (methylene) or 0.96 Å (methyl) and N—H = 0.86 Å, and were allowed to ride on their parent atoms, with *U*_iso_(H) = 1.2*U*_eq_(N, aromatic C or methylene C) or 1.5*U*_eq_(methyl C).

### Crystal data

**Table:**

C_10_H_11_N_3_O_2_	*Z* = 2
*M**_r_* = 205.22	*F*(000) = 216
Triclinic, *P*1	*D*_x_ = 1.295 Mg m^−^^3^
Hall symbol: -P 1	Mo *K*α radiation, λ = 0.71073 Å
*a* = 7.6320 (15) Å	Cell parameters from 25 reflections
*b* = 7.9200 (16) Å	θ = 9–13°
*c* = 9.2440 (18) Å	µ = 0.09 mm^−^^1^
α = 109.30 (3)°	*T* = 293 K
β = 91.28 (3)°	Block, yellow
γ = 93.00 (3)°	0.30 × 0.20 × 0.20 mm
*V* = 526.2 (2) Å^3^	

### Data collection

**Table:**

Enraf–Nonius CAD-4 four-circle diffractometer	1321 reflections with *I* > 2σ(*I*)
Radiation source: fine-focus sealed tube	*R*_int_ = 0.017
graphite	θ_max_ = 25.3°, θ_min_ = 2.3°
ω–2θ scans	*h* = 0→9
Absorption correction: ψ scan (North *et al.*, 1968)	*k* = −9→9
*T*_min_ = 0.973, *T*_max_ = 0.982	*l* = −11→11
2073 measured reflections	3 standard reflections every 200 reflections
1918 independent reflections	intensity decay: 1%

### Refinement

**Table:**

Refinement on *F*^2^	Secondary atom site location: difference Fourier map
Least-squares matrix: full	Hydrogen site location: inferred from neighbouring sites
*R*[*F*^2^ > 2σ(*F*^2^)] = 0.051	H-atom parameters constrained
*wR*(*F*^2^) = 0.165	*w* = 1/[σ^2^(*F*_o_^2^) + (0.10*P*)^2^0.002*P*] where *P* = (*F*_o_^2^ + 2*F*_c_^2^)/3
*S* = 1.04	(Δ/σ)_max_ < 0.001
1918 reflections	Δρ_max_ = 0.18 e Å^−^^3^
137 parameters	Δρ_min_ = −0.17 e Å^−^^3^
0 restraints	Extinction correction: *SHELXL97* (Sheldrick, 2008), Fc^*^=kFc[1+0.001xFc^2^λ^3^/sin(2θ)]^-1/4^
Primary atom site location: structure-invariant direct methods	Extinction coefficient: 0.22 (3)

### Special details

**Table:**

Geometry. All e.s.d.'s (except the e.s.d. in the dihedral angle between two l.s. planes) are estimated using the full covariance matrix. The cell e.s.d.'s are taken into account individually in the estimation of e.s.d.'s in distances, angles and torsion angles; correlations between e.s.d.'s in cell parameters are only used when they are defined by crystal symmetry. An approximate (isotropic) treatment of cell e.s.d.'s is used for estimating e.s.d.'s involving l.s. planes.
Refinement. Refinement of *F*^2^ against ALL reflections. The weighted *R*-factor *wR* and goodness of fit *S* are based on *F*^2^, conventional *R*-factors *R* are based on *F*, with *F* set to zero for negative *F*^2^. The threshold expression of *F*^2^ > σ(*F*^2^) is used only for calculating *R*-factors(gt) *etc*. and is not relevant to the choice of reflections for refinement. *R*-factors based on *F*^2^ are statistically about twice as large as those based on *F*, and *R*- factors based on ALL data will be even larger.

### Fractional atomic coordinates and isotropic or equivalent isotropic displacement parameters (Å^2^)

**Table:**

	*x*	*y*	*z*	*U*_iso_*/*U*_eq_	
N1	0.3192 (2)	0.1104 (2)	0.86877 (17)	0.0589 (5)	
H1A	0.3558	0.0076	0.8175	0.071*	
O1	0.2437 (2)	−0.30919 (19)	1.04167 (18)	0.0847 (6)	
C1	0.4572 (4)	0.3339 (4)	0.5821 (3)	0.0877 (8)	
H1B	0.5109	0.2853	0.4853	0.132*	
H1C	0.5341	0.4277	0.6502	0.132*	
H1D	0.3479	0.3816	0.5668	0.132*	
O2	0.3393 (2)	−0.22529 (19)	0.85827 (19)	0.0779 (5)	
N2	0.2712 (2)	−0.1930 (2)	0.98294 (19)	0.0586 (5)	
C2	0.4237 (3)	0.1875 (3)	0.6512 (2)	0.0720 (7)	
H2A	0.3477	0.0921	0.5811	0.086*	
H2B	0.5342	0.1378	0.6641	0.086*	
C3	0.3404 (3)	0.2542 (3)	0.8029 (2)	0.0631 (6)	
H3A	0.4132	0.3533	0.8722	0.076*	
H3B	0.2266	0.2975	0.7898	0.076*	
N3	−0.0667 (3)	0.2255 (3)	1.5411 (2)	0.0880 (7)	
C4	0.2472 (2)	0.1269 (2)	1.0023 (2)	0.0490 (5)	
C5	0.2198 (2)	−0.0143 (2)	1.0637 (2)	0.0489 (5)	
C6	0.1414 (2)	0.0104 (2)	1.2017 (2)	0.0539 (5)	
H6A	0.1264	−0.0853	1.2384	0.065*	
C7	0.0851 (3)	0.1755 (3)	1.2854 (2)	0.0547 (5)	
C8	0.1124 (3)	0.3178 (3)	1.2288 (2)	0.0599 (6)	
H8A	0.0770	0.4304	1.2853	0.072*	
C9	0.1890 (3)	0.2953 (2)	1.0940 (2)	0.0581 (5)	
H9A	0.2043	0.3931	1.0600	0.070*	
C10	0.0000 (3)	0.2015 (3)	1.4274 (3)	0.0661 (6)	

### Atomic displacement parameters (Å^2^)

**Table:**

	*U*^11^	*U*^22^	*U*^33^	*U*^12^	*U*^13^	*U*^23^
N1	0.0739 (11)	0.0428 (9)	0.0594 (10)	0.0047 (8)	0.0125 (8)	0.0154 (7)
O1	0.1188 (14)	0.0447 (8)	0.0985 (12)	0.0147 (8)	0.0172 (10)	0.0322 (8)
C1	0.105 (2)	0.0872 (17)	0.0765 (16)	−0.0142 (15)	0.0120 (14)	0.0368 (13)
O2	0.0994 (12)	0.0541 (9)	0.0783 (10)	0.0199 (8)	0.0273 (9)	0.0154 (7)
N2	0.0659 (11)	0.0401 (9)	0.0698 (11)	0.0078 (7)	0.0042 (8)	0.0175 (8)
C2	0.0830 (15)	0.0691 (14)	0.0656 (13)	0.0024 (12)	0.0124 (11)	0.0243 (11)
C3	0.0749 (14)	0.0539 (11)	0.0626 (13)	0.0001 (10)	0.0083 (10)	0.0222 (9)
N3	0.1119 (17)	0.0745 (13)	0.0812 (13)	0.0188 (12)	0.0356 (12)	0.0267 (10)
C4	0.0508 (11)	0.0416 (10)	0.0536 (11)	0.0000 (8)	0.0018 (8)	0.0149 (8)
C5	0.0497 (10)	0.0378 (10)	0.0575 (11)	0.0030 (8)	−0.0001 (8)	0.0136 (8)
C6	0.0583 (12)	0.0445 (10)	0.0620 (12)	−0.0004 (9)	0.0001 (9)	0.0226 (8)
C7	0.0566 (11)	0.0497 (11)	0.0566 (11)	0.0017 (9)	0.0055 (9)	0.0158 (8)
C8	0.0719 (13)	0.0414 (10)	0.0614 (12)	0.0063 (9)	0.0092 (10)	0.0098 (8)
C9	0.0702 (13)	0.0380 (10)	0.0669 (12)	0.0009 (9)	0.0059 (10)	0.0186 (9)
C10	0.0762 (14)	0.0556 (12)	0.0660 (14)	0.0056 (11)	0.0113 (11)	0.0186 (10)

### Geometric parameters (Å, °)

**Table:**

N1—C4	1.332 (2)	C3—H3A	0.9700
N1—C3	1.463 (2)	C3—H3B	0.9700
N1—H1A	0.8600	N3—C10	1.141 (2)
O1—N2	1.225 (2)	C4—C5	1.419 (2)
C1—C2	1.511 (3)	C4—C9	1.424 (3)
C1—H1B	0.9600	C5—C6	1.381 (2)
C1—H1C	0.9600	C6—C7	1.378 (3)
C1—H1D	0.9600	C6—H6A	0.9300
O2—N2	1.229 (2)	C7—C8	1.399 (3)
N2—C5	1.445 (2)	C7—C10	1.436 (3)
C2—C3	1.495 (3)	C8—C9	1.350 (3)
C2—H2A	0.9700	C8—H8A	0.9300
C2—H2B	0.9700	C9—H9A	0.9300
			
C4—N1—C3	124.78 (15)	C2—C3—H3B	109.6
C4—N1—H1A	117.6	H3A—C3—H3B	108.1
C3—N1—H1A	117.6	N1—C4—C5	124.93 (16)
C2—C1—H1B	109.5	N1—C4—C9	119.98 (16)
C2—C1—H1C	109.5	C5—C4—C9	115.09 (16)
H1B—C1—H1C	109.5	C6—C5—C4	122.25 (15)
C2—C1—H1D	109.5	C6—C5—N2	116.43 (15)
H1B—C1—H1D	109.5	C4—C5—N2	121.32 (16)
H1C—C1—H1D	109.5	C7—C6—C5	120.56 (16)
O1—N2—O2	121.83 (16)	C7—C6—H6A	119.7
O1—N2—C5	118.22 (16)	C5—C6—H6A	119.7
O2—N2—C5	119.95 (15)	C6—C7—C8	118.46 (17)
C3—C2—C1	112.3 (2)	C6—C7—C10	120.97 (17)
C3—C2—H2A	109.1	C8—C7—C10	120.57 (17)
C1—C2—H2A	109.1	C9—C8—C7	121.52 (17)
C3—C2—H2B	109.1	C9—C8—H8A	119.2
C1—C2—H2B	109.1	C7—C8—H8A	119.2
H2A—C2—H2B	107.9	C8—C9—C4	122.11 (17)
N1—C3—C2	110.26 (17)	C8—C9—H9A	118.9
N1—C3—H3A	109.6	C4—C9—H9A	118.9
C2—C3—H3A	109.6	N3—C10—C7	178.7 (2)
N1—C3—H3B	109.6		
			
C4—N1—C3—C2	−179.22 (17)	O2—N2—C5—C4	−0.4 (3)
C1—C2—C3—N1	−177.02 (18)	C4—C5—C6—C7	0.4 (3)
C3—N1—C4—C5	177.27 (18)	N2—C5—C6—C7	−178.84 (17)
C3—N1—C4—C9	−1.8 (3)	C5—C6—C7—C8	−1.2 (3)
N1—C4—C5—C6	−178.73 (16)	C5—C6—C7—C10	178.64 (17)
C9—C4—C5—C6	0.4 (3)	C6—C7—C8—C9	1.2 (3)
N1—C4—C5—N2	0.5 (3)	C10—C7—C8—C9	−178.65 (18)
C9—C4—C5—N2	179.62 (16)	C7—C8—C9—C4	−0.4 (3)
O1—N2—C5—C6	−0.5 (3)	N1—C4—C9—C8	178.75 (17)
O2—N2—C5—C6	178.87 (16)	C5—C4—C9—C8	−0.4 (3)
O1—N2—C5—C4	−179.81 (17)		

### Hydrogen-bond geometry (Å, °)

**Table:**

*D*—H···*A*	*D*—H	H···*A*	*D*···*A*	*D*—H···*A*
N1—H1A···O2	0.86	2.00	2.641 (2)	131
N1—H1A···N2	0.86	2.61	2.937 (2)	104
C6—H6A···O1	0.93	2.32	2.644 (2)	100
C9—H9A···O1^i^	0.93	2.42	3.331 (2)	165

Symmetry codes: (i) *x*, *y*+1, *z*.
